# Nurses’ Work Methods Assessment Scale: a study of content validation

**DOI:** 10.1590/0034-7167-2022-0396

**Published:** 2023-02-06

**Authors:** João Miguel Almeida Ventura-Silva, Maria Manuela Ferreira Pereira da Silva Martins, Letícia de Lima Trindade, Ana da Conceição Alves Faria, Sónia Cristina da Costa Barros, Ricardo Manuel da Costa Melo, Elaine Cristina Novatzki Forte, Olga Maria Pimenta Lopes Ribeiro

**Affiliations:** I Universidade do Porto, Instituto de Ciências Biomédicas Abel Salazar. Porto, Portugal; II Escola Superior de Enfermagem do Porto. Porto, Portugal; III Universidade do Estado de Santa Catarina. Chapecó, Santa Catarina, Brazil; IV Centro Hospitalar Universitário São João. Porto, Portugal; V Escola Superior de Saúde Norte da Cruz Vermelha Portuguesa. Oliveira de Azeméis, Portugal; VI Universidade Federal de Santa Catarina. Florianópolis, Santa Catarina, Brazil

**Keywords:** Validation Studies, Nursing Care, Work, Female and Male Nurses, Nursing Administration Research, Estudios de Validación, Atención de Enfermería, Trabajo, Enfermeras y Enfermeros, Investigación en Administración de Enfermería, Estudos de Validação, Cuidados de Enfermagem, Trabalho, Enfermeiras e Enfermeiros, Pesquisa em Administração de Enfermagem

## Abstract

**Objective:**

To develop and validate the content of the Nurses’ Work Methods Assessment Scale.

**Methods:**

Methodological study conducted between January and February 2022, based on the Quality Standards for Nursing Care and Imogene King’s framework. A literature review was previously undertaken to design the scale. The content validation was carried out by 23 experts.

**Results:**

The initial version consisted of 40 items organized in four work methods identified in the literature. In the first dimension, from ten items, seven were considered, and one was reformulated. Seven of the initial ten items were considered in the second version. The third dimension consisted of seven items. In the fourth dimension, three items were reformulated, and three were excluded, leaving seven items. The final version ended with 28 items, whose Content Validity Index ranged between 0.83 and 1.

**Conclusions:**

The involvement of experts has become pivotal in the development and validation of the items, providing confidence to the continuity of psychometric procedures.

## RESUMO

**Objetivo:**

Construir e validar o conteúdo da Escala de Avaliação dos Métodos de Trabalho dos Enfermeiros.

**Métodos:**

Estudo metodológico realizado entre janeiro e fevereiro de 2022, baseado nos Padrões de Qualidade dos Cuidados de Enfermagem e no referencial de Imogene King. Para construção da Escala, realizou-se previamente revisão de literatura. A validação de conteúdo contou com 23 peritos.

**Resultados:**

A versão inicial continha 40 itens, organizados nos quatro métodos de trabalho identificados na literatura. Na primeira dimensão, dos dez itens, foram considerados sete e um foi reformulado. Na segunda, dos dez iniciais, foram considerados sete. A terceira dimensão constituiu-se por sete itens. Na quarta dimensão, reformularam-se três e excluíram-se três, ficando sete. A versão final ficou com 28 itens, cujo Índice de Validade de Conteúdo oscilou entre 0,83 e 1.

**Conclusões:**

Recorrer aos peritos tornou-se fundamental na construção e validação dos itens, conferindo segurança à continuidade dos procedimentos psicométricos.

## RESUMEN

**Objetivo:**

Construir y validar contenido de Escala de Evaluación de Métodos de Trabajo de Enfermeros.

**Métodos:**

Estudio metodológico realizado entre enero y febrero de 2022, basado en Estándares de Calidad de Atención de Enfermeríay referencial de Imogene King. Para construcción de Escala, realizado previamente revisión de literatura. La validez de contenido contó con 23 peritos.

**Resultados:**

La versión inicial contenía 40 items, organizados en cuatro métodos de trabajo identificados en la literatura. La primera dimensión, de diez items, fueron considerados siete y uno fue reformulado. La segunda, de diez iniciales, fueron considerados siete. La tercera dimensión constituída por siete ítems. La cuarta dimensión, reformulados tres y excluidos tres, quedando siete. La versión final quedó con 28 items, cuyo Índice de Validez de Contenido osciló entre 0,83 y 1.

**Conclusiones:**

Recorrer a peritos volvió fundamental en la construcción y validez de los items, confiriendo seguridad a continuidad de procedimientos psicométricos.

## INTRODUCTION

In the last decades, one of the health organizations concerns worldwide has been focused on the quality of care and patient safety. The results of some authors’ works over time have brought to light, in the healthcare field, the presence of increasingly exigent people, the existence of complex job dynamics, and a rapidly developing technology, facing an increasingly globalized world and reflecting the practice of each professional^(1, 2)^. In this context, the World Health Organization reports that the domains of care safety and quality have improved with the implementation of evidence-based guidelines with proven effectiveness^(3)^.

Within the scope of nursing care, nurses have always been at the forefront of care delivery from a perspective of designing and executing quality, safe and accessible care, given the innovations and advances that science has brought to healthcare^(4)^.

In 2001, the *Ordem dos Enfermeiros of Portugal* pointed out the need for professionals to guide their professional practice based on the Nursing Care Quality Standards. That regulatory instrument shows society what to expect of nursing care, what nurses expect as a body, and what each one should do for a quality professional practice^(5, 6)^. The same regulatory institution presents six descriptive statements to achieve those standards, which allow the orientation of nurses’ professional practice and consideration of the care provided, decision-making in nursing, and definition of the nurse’s role when dealing with patients, other professionals, political decision-makers, and society in general^(6)^. In the instrument stood out patient satisfaction; health promotion; prevention of complications; well-being and self-care; functional readaptation; and organization of nursing care.

Regarding the organization of nursing care, nurses need to adopt methodologies that promote quality^(5)^ and ensure the safety of the care provided. In an international study, the authors found that nurses identified that the organizational methodology of nursing care utilized in their service mainly promoted quality, and only a minority disagreed with that opinion^(7)^.

A work method describes nurses’ independent or cooperative approaches to providing direct care and meeting patient needs^(8,9)^. Over time, the work methods have evolved according to innovation and technology, as well as patients’ demands for quality care; those methods have traditionally been conceptualized as functional work methods, individual work methods, teamwork methods, and reference nurse work methods^(10)^. However, they may vary according to philosophy, design, workload, nursing staff, patient organization, skill mix, and cost, but they aim to improve the quality of nursing care^(11)^. The choice of a work method expresses a perspective, a philosophy of care, a way of thinking, and organizing nurses and the nursing team care, which will be decisive for patient safety and the quality of nursing care^(10, 11)^.

In a recent review, the authors confirmed that the use of a work method has implications for the patient, the nurses themselves, and the institutions, allowing to show the nurse’s work and formative path and the institutional characteristics of the different contexts of care^(10)^.

Given the importance of that issue for nurses’ professional practice and in light of the literature on how nurses organize their work, the development of an instrument became urgent. For this purpose, we applied Imogene King’s Goal Achievement Theory. According to the author, nursing is dedicated to the study of caring for human beings by addressing the needs observed by patients and nurses, trying to evaluate the opportunities of self-care, and exploring the means to achieve a goal^(12)^. In this regard, King demonstrated concern with the very essence of nursing, understanding that how nurses practice and care for people throughout their professional performance differentiates nursing from other health professions. The author also demonstrated the importance of nurses adopting a work method and a record system that ensures data collection, problem identification, implementation, and evaluation of the effectiveness of nursing care^(12, 13)^.

## OBJECTIVE

To develop and validate the contents of the Nurses’ Work Methods Assessment Scale.

## METHODS

### Ethical aspects

The development of this study followed the legal and ethical principles recommended by the National and International Ethics Standards for research involving human beings. All participants were informed about the objectives and purpose of the study and signed an informed consent form, with a commitment from the investigators to guarantee confidentiality and anonymity while using the information collected. This study was based on the broader research study “Nurses’ Working Methods in the Hospital Environment: Contributions to the Quality and Safety of Care,” approved in February 2022 by an Ethics Committee under No 421-21.

### Design, period, and place of study

This is a methodological study for the development and content validation of an instrument to evaluate the work methods of nurses in a hospital environment.

The study was conducted in a leading hospital institution located in northern Portugal. In agreement with the Quality Standards for Nursing Care^(6)^, it is noteworthy that this institution has invested in methodologies for organizing nursing care.

Data were collected with the experts between January and February 2022 through an electronic questionnaire via Google Forms. Besides the proposal of the several items to be included in the Nurses’ Work Methods Assessment Scale (*Escala de Avaliação dos Métodos de Trabalho dos Enfermeiros* - EAMTE), it was also performed a sociodemographic and professional definition of the experts.

### Sample, criteria of inclusion and exclusion

A non-probability purposive sample was applied to select the experts. The inclusion criteria were professional nurses, nurse specialists, nurse managers, and nursing teachers with a professional practice of six months or more who agreed to participate in the study. From 29 selected experts, we obtained 23 responses (79.3%). The invitation to participate was sent by email, noting that, before filling out the questionnaire, the experts had to give their free and informed consent to participate.

### Study protocol

In the initial phase, the items included in the instrument were initially designed using a scoping review to map the scientific evidence on nurses’ work methods in a hospital environment. The Quality Standards for Nursing Care^(6)^ from the Portuguese Nurses’ Association (namely the descriptive statement “Organization of Nursing Care”) and Imogene King’s Theory of Goal Achievement^(12)^ were the applied theoretical references. Consequently, we organize the findings according to each type of work method associated with nurses’ professional performance^(10)^: functional work method, individual work method, teamwork method, and reference nurse work method.

After that stage, the first version of the Nurses’ Work Methods Assessment Scale (EAMTE), consisted of 40 items: 10 items for each work method.

The content validation of the items included in the instrument was performed using expert (judges) analysis presented in this study. In this phase, to assess each item on the scale, a Likert-type scale graded in three points was used^(14)^: disagree; nor disagree/neither agree; agree, considering three criteria: relevance to the construct, clarity, and similarity to other items. In addition to the previous criteria, each expert could give their opinion about the need for reformulation, exclusion, or repositioning.

### Analysis of results and statistics

Regarding each item’s evaluation criteria, we always maintained the statement when relevance and clarity were greater than 80%. In case of similarity with other items, we removed them. The Content Validity Index (CVI)^(14, 15)^ estimation verified the experts’ agreement about the representativeness of each item with the addressed content. Thus, if the CVI was ≥ 0.80, the item in the analysis was valid and should be included in the instrument^(15)^. The estimate of each item’s CVI resulted from the division of the number of experts who agreed with the item by the total number of professionals^(14)^.

## RESULTS

Twenty-three experts participated in the study: 69.6% nurses, nurse specialists or nurse managers, and 30.4% nursing faculty members, primarily female (69.6%), married or living in a stable union (78.3%). The mean age was 43.5 years, with a standard deviation of 9.6, with a minimum age of 30 and a maximum age of 62. Regarding the academic degree, 30.4% had a bachelor’s degree, 39.1 % had a master’s degree, and 30.5% had a doctoral degree. As for the profession, 21.8% were nurses; 26.1% were nurse specialists; 21.8% were nurse managers; 26.1% were assistant professors; 4.3% were professor coordinators. The average time of professional practice was 21.5 years, standard deviation of 10.2, ranging from 7 to 41 years.

The questionnaire submitted to expert validation was comprised of 40 items distributed into four dimensions corresponding to the four work methods adopted by nurses and identified in the literature review^(10)^: functional work method, individual work method, teamwork method, and reference nurse work method.

As a result of the assessment of the Functional Work Method dimension, seven of the ten items proposed were considered adequate, but one needed to rewrite. The repetition of information, the confusing writing, and the presence of aspects difficult to quantify determined the exclusion of three items. In order to understand the pertinence of each item to the construct, we calculated the CVI of the ten items, whose results are presented in Table 1.

**Table 1:** Experts’ agreement regarding the representativeness of the construct of the items of the Functional Work Method dimension

Items of the “Functional Work Method” dimension	CVI
1. During my shift, I perform nursing interventions on all patients admitted to the service.	0.91
2. When patients are admitted, I divide the interventions with the rest of the nurses to facilitate the work.	0.87
3. The focus of my work is centered on the execution of predefined and standardized procedures.	0.91
4. I feel that my work is recognized through standardized interventions.	0.91
5. During the planning and implementation of interventions, I devote myself exclusively to their execution without the patient’s involvement in the care.	0.91
6. During the planning and implementation of the interventions, I dedicate myself exclusively to their execution, without the involvement of the caregiver/family member caregiver.	1.00
7. The outcome of the nursing care I provide aims to deliver the interventions.	0.83

From the experts’ analysis, seven out of ten items were considered adequate in the Individual Work Method dimension. The absence of relevant content and the repetition of information determined the exclusion of three items. Thus, the second version of the dimension was composed of seven items. Table 2 highlights the relevance of each item of the Individual Work Method, according to the CVI.

**Table 2:** Experts’ agreement regarding the representativeness of the construct of the items of the Individual Work Method dimension

Items of the “Individual Work Method” dimension	CVI
1. During a work shift, I take full responsibility for formulating and executing care for the patients assigned to me.	0.87
2. My work focuses on the conception and implementation of patient care during a shift.	0.87
3. In the organization of nursing care, I value the integration of the patient over the execution of standardized interventions.	0.96
4. I believe that monitoring the same patients throughout a shift ensures more humanized and personalized nursing care.	0.91
5. The organization of the nursing care that I provide aims at the integral care of the patient.	0.91
6. In the planning and implementing interventions, I consider the involvement of the family/family member caregiver.	0.83
7. In each work shift, I evaluate the results of the interventions to reformulate the care planning.	0.96

Regarding the Teamwork Method dimension, of the ten items proposed for expert analysis, three were excluded due to repetition and irrelevant information, which is why seven items were considered adequate. One item was reformulated due to elements related to syntax. Table 3 shows the CVI calculation for each item of the Teamwork Method.

**Table 3:** Experts’ agreement regarding the representativeness of the construct of the items of the Teamwork Method dimension

Items of the “Teamwork Method” dimension	CVI
1. The nursing care I provide is guided and supervised by a nurse designated as team leader/chief of staff.	0.91
2. Daily meetings between the nurses and the nurse leaders/team leaders discuss the nursing care to be provided to the patient to ensure continuity of care.	0.87
3. There is a team leader responsible for ensuring quality and safe nursing care, which employs leadership strategies, control, and supervision techniques.	0.87
4. I value the continuity of care by discussing nursing care plans between nurse leaders/team leaders and the remaining nurses.	0.91
5. Patients’ care needs are met by a team of nurses with different levels of competence.	1.00
6. Throughout each shift, the planning and implementation of care to be performed by a group of nurses are common.	0.96
7. The team leader discusses strategies with the nurses to involve the family/family member caregiver in the planning and implementing of care.	0.87

In the Reference Nurse Work Method dimension, the final version of the Nurses’ Work Methods Assessment Scale (EAMTE), also included seven items. Before the experts’ assessment, there were ten items. After their analysis, three items were excluded, and three activities had to be reformulated for better understanding. Based on the CVI, Table 4 shows the values of each item of the Reference Nurse Work Method dimension.

**Table 4:** Experts’ agreement regarding the representativeness of the construct of the items of the Reference Nurse Work Method dimension

Items of the “Reference Nurse Work Method” dimension	CVI
1. There is a reference nurse for each patient, from admission to discharge, and other nurses who substitute in their absence.	0.91
2. In the absence of the reference nurse, assistance will be provided by nurses “associated” with them.	0.91
3. Whenever possible, I am assigned the patients I admitted to the service.	0.96
4. The patient’s reference nurse plans and evaluates the care provided by themselves and associate nurses, suggesting changes in the planning and implementation of care from admission to discharge.	0.91
5. The reference nurse validates the reformulation of care planning.	0.87
6. I always try to include the patient during the planning and implementation of interventions.	0.91
7. During the planning and implementation of nursing interventions, I always try to include the caregiver/family member caregiver.	0.83

## DISCUSSION

The organization of nursing care forms a fundamental aspect of nursing care that guarantees effective benefits for the patient, the nurse, and the institution^(10, 16)^. The literature points out that the selection of a work method by nurses may enhance patient safety and the quality of nursing care^(10)^.

Several authors in previous studies have evidenced that nurses’ work methods have evolved over the years, following the various demands imposed by the provision of quality care. It is possible to identify four work methods adopted by nurses: functional work method, oriented towards task execution; teamwork method, focused on the patient; work method, focused on total patient care, also called individual; and, finally, the reference nurse work method or Primary Nursing^(10, 17)^.

However, given the importance of this issue to the nurses, in the national and international contexts, instruments could not be found that allowed which nursing activities to be part of each work method. Thus, the challenge was to develop an instrument to reflect how nurses organize their work, give social visibility to what nurses do, and contribute to the advancement of nursing and professional practice.

It should be noted that this research allowed for the content validation of the Nurses’ Work Method Assessment Scale, with the intervention of experts who had the opportunity to suggest some changes regarding the relevance and clarity of each item. Thus, the instrument was adapted to the context of clinical practice.

Selecting experts with experience in the academic environment and clinical practice was intended to contribute to a more reliable instrument. Thus, it was possible to combine aspects of clinical practice with those of the academic context and to rigorously select the items included in each dimension, consistent with the safety and quality of nursing care.

In the developed instrument in 2001 - following the orientation of the *Ordem dos Enfermeiros de Portugal,* reviewed and re-published in 2012^(6)^ - and after the literature review, four dimensions were defined, related to each work method.

In the Functional Work Method dimension, the seven items of the current scale version focused on a nursing practice directed to accomplishing tasks, separating the planning and execution and detachment from the patient and family throughout the care process. The focus of this method turns to the distribution of work based on the performance of tasks and procedures, in which the target of action is not the patient but the task^(9, 10)^.

In 2021, a study analyzed the nurses’ work methods by employing nursing and management theories. The authors pointed out that nursing care using that method reached proficiency through systematic repetition of techniques such as drug administration, monitoring vital signs, and provision of hygiene care. There was a lack of coordination among them, represented by “fragmented interventions”^(11)^. This issue was considered in the creation of the activities. It is also important to highlight the difficulty in including the nursing process, given the philosophy of a fragmented practice, which corroborates previous literature^(10, 11, 18)^.

Seven items of the Individual Work Method dimension highlight the patient-oriented work methods, conceived according to the patient’s integrated care, emphasizing individual care, integrated treatment, and satisfying the patient’s needs. The literature points out that a single nurse is responsible for planning and executing integrated care for one or more patients, meeting their needs^(18)^. Effectively, the concern with the involvement of the patient and family member caregiver throughout the care process is verified, making them participants^(11)^. Furthermore, it is necessary to use the nursing process to identify and meet the patient’s needs for person-centered care and sustained decision-making^(19)^, continuity of care^(11)^, and increased safety and quality of nursing care^(20)^.

The Teamwork Method dimension of the current version of the Nurses’ Work Methods Assessment Scale (EAMTE), after the experts’ evaluation, has seven items that reflect the organization of the nurses’ work regarding teamwork, focusing on the presence of a team leader, integrated patient care, the planning of care and team decision-making, as well as the participation of the patient and family member caregiver in the whole process. In an international study, it was evident that all nurses know the patient’s needs and/or problems, assisting them in their entirety and contributing in a particular way to their well-being^(21)^. Furthermore, the literature points out that nurses are divided into teams and guided by team leaders, which besides constituting a reference for nurses, allows them to assume the responsibility of maximizing the group’s capabilities and the different individual competencies of each nurse^(21, 22)^. These aspects are taken into consideration in the development of the activities of this dimension.

In the Reference Nurse Work Method dimension, the focus was on the definition of a nurse as responsible for the patient care process from admission until clinical discharge. In reality, the seven items that are part of the dimension emphasize the importance of integrated care, the responsibility of a nurse, and the nurses associated with them regarding the relevant data gathering, planning, and execution of nursing interventions, with the participation of the patient and family member caregiver. Those items also highlight the reformulation of the care plan according to the patient’s problems, ensuring aspects associated with the safety and quality of nursing care.

In a study evaluating the association between the implementation of the reference nurse work method and the reduction of omissions in nursing care, it was clear that omitted nursing care reduced by 78.5%; this was reflected in increased satisfaction with the nursing team and was made possible by more involvement with the patient and family member caregiver, more accurate identification of care needs, planning and implementation of nursing interventions and, as such, increased safety of nursing care^(23)^. In another study, which analyzed the impact of the reference nurse work method on nursing care and the accuracy of nursing recordkeeping, the authors found that the way nurses documented care reflected the nursing process, especially the actual problems of the patient and the evaluation of nursing interventions to solve those problems. Thus, emphasis was placed on patient satisfaction with the care provided, the quality of nursing care, and the nurse’s competencies^(24)^, which are consistent with the items of the scale defined for this dimension.

Regarding the values obtained in the CVI, although the EAMTE revealed adequate content validity, we emphasize that this study refers to one of the first phases of instrument development. Therefore, we will need to apply this instrument to the target population with psychometric procedures already being carried out in a multicenter study. It is also relevant to highlight that the EAMTE was developed based on the professional practice of nurses and nurse specialists, following both the Nursing Care Quality Standards recommended by the *Ordem dos Enfermeiros of Portugal and* Imogene King’s Goal Achievement Theory. In addition, the experts’ intervention was essential to validate the items’ relevance, improve the activities’ syntax and writing and, thus, adapt the instrument.

### Study limitations

New instruments that provide evidence on nurses’ work methods allow for monitoring practices and improving the quality of care. In this respect, the circumstances (region of the country, temporality, among others) of the validation are a limit, being appropriate to validate the instrument in their research scenarios.

### Contributions to the field

The 28 items of the current version of the Nurses’ Work Methods Assessment Scale (EAMTE), focus on the activities that might integrate the four work methods which are adopted by nurses in their professional practice. Those items are important indicators of how each nurse organizes their own work, providing nurses’ subsidies for social visibility and the maintenance of nursing services within a logic of providing safe and quality care.

## CONCLUSIONS

The content validation of the Nurses’ Work Methods Assessment Scale (EAMTE) was a way to confirm the theoretical relevance of each item included in the four dimensions of the scale. The steps taken so far made it possible to highlight the instrument’s purpose and emphasize the need for the continuity of the scale’s creation/development process regarding the procedures inherent to validation. The EAMTE), will enable the perception of how nurses organize their work, especially nursing care management, which is expected to be safe and high-quality.
